# Genetic Mapping and Validation of Quantitative Trait Loci (QTL) for the Grain Appearance and Quality Traits in Rice (*Oryza sativa* L.) by Using Recombinant Inbred Line (RIL) Population

**DOI:** 10.1155/2019/3160275

**Published:** 2019-02-25

**Authors:** Leila Bazrkar-Khatibani, Barat-Ali Fakheri, Maryam Hosseini-Chaleshtori, Anumalla Mahender, Nafiseh Mahdinejad, Jauhar Ali

**Affiliations:** ^1^Plant Breeding and Biotechnology Department, Zabol University, Iran; ^2^Rice Research Institute of Iran (RRII), Agricultural Research, Education and Extension Organization, Rasht 1658, Iran; ^3^Rice Breeding Platform, International Rice Research Institute (IRRI), Los Baños, Laguna 4031, Philippines

## Abstract

Rice grain shape and nutritional quality traits have high economic value for commercial production of rice and largely determine the market price, besides influencing the global food demand for high-quality rice. In order to understand the genetic components of grain appearance traits in paddy, brown, and head rice, 15 traits were evaluated by using 157 recombinant inbred lines (RILs) derived from a cross between two Iranian rice cultivars Ali-Kazemi (A) and Kadous (K). A significant variation was observed and showed transgressive segregation among the RILs. Correlations between the visual appearances of grain traits were studied. A linkage map with 65 polymorphic SSR markers was constructed, which covered 1517.32 cM of the rice genome. A total of seven QTLs were identified on four chromosomes, 1, 6, 9, and 12, associated with four traits, which are explained by the total phenotypic variation of 44.27% and LOD score of 32.77 in 2014 and 2015, respectively. Among these, four QTLs for two traits were consistently flanked by RM23904 and RM24432 on chromosome 9. Single QTL for head grain length (HGL) expressed in both the years on chromosomes 1 and 9. A major QTL for seed weight was detected on chromosome 9, which explained 10.18% of the phenotypic variation. The additive effect of all the QTLs was positively contributed by Ali-Kazemi allele, except one QTL on chromosome 6 (*qBGL_6*) that showed a negative additive effect being contributed by the Kadous allele. The study also validated the identified QTLs with the polymorphic SSR markers that were previously reported. Novel QTLs were identified on chromosomes 6 and 9, and many of the polymorphic markers were found to be associated with milling processing of grain quality, cooking, and nutraceutical properties of rice by extensive literature and database analysis. Therefore, these validated QTLs and marker information could be utilized in the marker-assisted selection to improve grain appearance and nutritional grain quality traits in rice.

## 1. Introduction

Rice (*Oryza sativa* L.), is one of the major staple food crops for more than 3.5 billion global population. The production and consumption of global rice accounted for almost 90% by Asian countries; mainly China and India alone contribute about 55% [1]. Among the cereals, rice provides up to 20% of their regular calorie intake for millions of global population. In order to ensure nutritional food security, the projected rice production must be increased to 852 million tons by 2035 [2]. With the intensifications of diverse food demands and living standards of global populations, rice grain appearance and quality have become a primary concern for rice breeders. Therefore, there is an urgent need to increase the grain yield along with desirable grain nutritional quality (GQ) traits in rice [3, 4]. The rice grain attributed traits are a major contributor to the determination of the acceptability by end users and market price [5, 6]. GQ has been recognized into two classes: (i) grain appearance and (ii) cooking and eating qualities [7]. The grain appearance determined by the length, breadth, length-breadth ratio, endosperm translucency, and cooking and eating quality traits includes aroma, volume expansion, cooked kernel elongation, firmness/stickiness (related to amylose content), gelatinization temperature (measured as the alkali spreading value), and mouthfeel [8, 9]. The GQ traits are known to be associated with physical appearance of grain size with several aspects of grain quality attributes related to cooking, nutritional, and sensory characteristics [10, 11].

Globally, the rice producers' and consumers' major concern is grain appearance which also contributes to the reputation of a given variety. Although the preferences for rice grain characteristics vary with different consumer groups, long and slender rice is usually preferred by many consumers in India, Vietnam, USA, and most of the Asian countries [7, 12], whereas short and round rice grain is consumed in Northern China, Korea, and Japan. The long-grained rice varieties tend to produce dry, fluffy, and separated grains after being cooked, whereas the medium and short grain varieties tend to produce clumped, moist, and chewy grains after cooking [13]. The GQ trait inheritance in rice is complex that includes many components. Among them, amylose content (AC), gelatinization temperature (GT), gel consistency (GC), and protein content (PC) have been extensively studied and found to be controlled by QTLs with major and minor effects [9].

The milling yield or milling efficiency is another major factor to determine the percentage of the whole kernel milled rice obtained from rough rice (paddy rice) after milling, and the price of whole grain is typically twice as that of broken grains [14]. Therefore, it is an economically important trait. During milling processes, it combines multiple steps such as hulling and removal of the bran and embryo, followed by separating whole grains from broken grains. This gives rise to three milling components: brown rice ratio, milled rice ratio, and head rice ratio [15]. Another component of this process yields rice bran, an essential source of protein, vitamins, minerals, antioxidants, and phytosterols [16]. Rice bran protein has immense potential in the food industry, having unique nutraceutical properties and reported as a hypoallergenic food ingredient in infant formulations and anticancer properties [17].

With the increase in literacy percentage and awareness about diets, the people showed a tendency to be more health conscious and interested to take nutritionally enriched food. The quality of rice is an important character to determine the economic value in the export market and consumer acceptance [11]. The preference for rice quality varies across regions, as Japanese people prefer short grain and sticky rice [18]. Conversely, the whole northern part of India, Pakistan, and the Middle East regions prefer Basmati rice. The appearance of GQ has been determined by grain length, width, the length-width ratio (LWR), chalkiness of the endosperm, and grain shape, and size has a direct effect on the marketability and consumer acceptance [19]. The rice consumers' preference for grain shape differs from one location to another. Therefore, grain shape trait needs to be understood and considered in the context of market preference of the region and breeding approaches. The physical appearance of rice grain such as the shape and width greatly influences other vital rice quality traits, viz., endosperm chalkiness, milling efficiency, and cooking and eating properties [20]. Identification and development of superior GQ traits by direct examination in the field are very difficult due to trait complexity and being easily influenced by the environment [21, 22]. Therefore, with the recent development of DNA molecular marker technology and physical linkage map of rice, it has become feasible to dissect complex polygenic traits in rice. Thus, there is a need to understand the genetic basis of GQ traits and develop suitable strategies to develop the consumer preferred type of quality rice.

In a corollary of classical plant breeding methods, with the intervention of molecular markers and advanced genomic techniques, existing varieties could be improved to target the most vulnerable people of developing nations from micronutrient deficiency [9, 10]. The major targets to enhance rice GQ include appearance, milling, cooking, and physicochemical properties and nutritional qualities in rice. Many of these traits are highly complex governed by quantitative trait loci (QTLs) that are influenced by environmental factors and showed genetic variation to be polygenic in segregating progeny of intervarietal crosses, natural populations as landraces, and among inbred lines. The efficiency of molecular markers for the improvement of grain appearance and nutritional quality traits has been successfully utilized in various genetic backgrounds, mapping populations, and landraces of rice genotypes [10, 23–27]. Among the various types of molecular markers, SSR, STS, and SNP markers are highly useful when they are located within a gene of interest or in linkage disequilibrium with the gene throughout a population.

In the last several years, many QTLs for grain appearance and eating quality have been identified from different mapping populations: RILs, DHs, CSSLs, F_2_, and BC_2_F_2_ (reviewed by Mahender et al. [9]). Over the past two decades, many QTLs associated with grain dimension have been mapped in several populations of different chromosomes in rice. From the comprehensive literature survey and Gramene database, we found a significant number of QTLs distributed on different chromosomes. A total of 59 QTLs for MPGQ, 71 QTLs for GA, 36 QTLs for CP, seven QTLs for FRG, and 86 QTLs for NF were identified to date (Supplementary Table 1). Another critical trait is grain chalkiness (GCH), which influences the grain quality and market price. About 82 QTLs related with GCH traits, including 30 QTLs for percentage grain with white core (PGWC), 26 QTLs for degree of endosperm chalkiness (DEC), 12 QTLs for area of endosperm chalkiness (AEC), 11 QTLs for white-backed kernel, and 3 QTLs for basal white (BW), were reported [28, 29] in the Gramene database. Among them, the majority of the QTLs with ≥3 LOD score were located on chromosome 1 [5, 30], chromosome 2 [30, 31], chromosome 3 [21, 32–34], chromosome 4 [35, 36], chromosome 5 [37, 38], chromosome 6 [32, 36], chromosome 7 [5, 30, 38, 39], and chromosome 10 [37]. Up to date, a total of 14 major genes were significantly involved in controlling the grain shape and size, totally, four genes (*GW2*, *GW8*, *GS5*, and *GW5/qSW5*) for grain width, eight genes (*GS2/GL2*, *GL3.1/qGL3*, *GL4*, *GLW7*, *GS3*, *OsLG3*, *OsLG3b/qLGY3*, and *TGW6*) for grain length, and two genes (*GW6a* and *GL7/GW7*) for similar effects on grain length, width, and also in larger impact on grain weight [40, 41]. Despite these QTL mapping efforts, only limited information is available for a combination of the physical appearance of paddy, brown, and head rice grain visual quality traits and associated QTL information. Based on the comprehensive information of grain appearance and quality traits, associated QTLs, in the present study, were investigated by using recombinant inbred lines (RILs) derived from a cross between *indica* species of two Iranian rice cultivars such as Ali-Kazemi which is a native rice cultivar from Guilan Province of northern Iran and improved rice cultivar of Kadous. Interestingly, in more than 60% of rice-growing areas in Iran, farmers have been using many traditional and local rice varieties for their primary concern to grain nutritional and cooking quality. However, the genetic analyses of this background of parents and RILs have not been explored. Therefore, the present study was carried out by considering the following objectives: (i) to evaluate grain appearance traits and their correlations, (ii) to identify the QTLs by using composite interval mapping method, and (iii) to validate the QTL-linked molecular markers with previously reported QTLs.

## 2. Materials and Methods

### 2.1. Plant Materials

Parental lines, *Oryza sativa ssp. indica* cv. Ali-Kazemi which is a local rice cultivar and Kadous which is an improved Iranian rice variety, were used for the development of mapping population to identify genomic region conferring grain appearance traits. The native rice cultivar of Ali-Kazemi is adapted to the northern region of Iran, and it possesses better grain and cooking quality traits (grain yield: 3.8-4 t ha^−1^; amylose content: 16.7; gelatinization temperature: 4.9; gel consistency: 70; the average length of grain before cooking: 7.2 mm, after cooking: 11.6 mm; width 2.1-2.3 mm; and the elongation ratio: 1.6). The improved rice cultivar of Kadous provides high grain yield and their grain quality traits as grain yield: 6.5-7 t ha^−1^; amylose content: 23.25; gelatinization temperature: 3.3; gel consistency: 52; the average length of grain before cooking: 7.8 mm, after cooking: 10.25 mm; width ≤ 2 mm; and the elongation ratio: 1.3 [42]. This improved rice variety was developed from two International Rice Research Institute (IRRI) lines IR67015-94-2-3 and IR64669-153-2-3, respectively. A total of 157 RILs in F_9_ generation were developed by using single seed descent (SSD) [43]. The RILs and parents were planted during the rice-growing seasons of 2014 and 2015 in the experimental farms of the Rice Research Institute of Iran (RRII) located in the northwestern area of Rasht in Guilan, Iran. The F_9_ plants and parents were raised in a randomized complete block design with three replications with a spacing of 25 × 25 cm. Field's management followed the normal agricultural practices.

### 2.2. Phenotyping

Mature F_9_ grains of the RILs and parental lines were air-dried and stored at room temperature (37-40°C) for at least two months before milling. The moisture content of each sample was reduced to 14% for both years. The length and width of white rice were measured by a photoenlarger, and the length, width, and thickness of paddy and brown rice were measured using digital Vernier calipers (0-150 mm). Meanwhile, the length-to-width ratios were estimated using the obtained data. The percentage of chalkiness was also determined by counting the number of chalky grains selected from 100 grains for each line. A total of 15 grain appearance traits in paddy, brown, and head rice were followed by paddy grain length (PGL), paddy grain width (PGW), paddy grain thickness (PGT), paddy grain length-to-width ratio (PGLWR), paddy grain length-to-thickness ratio (PGLTR), brown grain length (BGL), brown grain width (BGW), brown grain thickness (BGT), brown grain length-to-width ratio (BGLWR), brown grain length-to-thickness ratio (BGLTR), head grain length (HGL), head grain width (HGW), head grain length-to-width ratio (HGLWR), seed weight (SW), and chalkiness (CH) and were recorded. Further, phenotypic trait investigation was followed by descriptive statistical analysis which are the mean, range, standard deviation, the coefficient of variation, skewness, and kurtosis information which were calculated by using STAR software tool version 1.4 package (http://bbi.irri.org) and MS Excel. Also, the Pearson correlation coefficient was estimated between the phenotypic traits by using the *corrplot* package in R software (https://github.com/taiyun/corrplot).

### 2.3. Genotyping by SSR Markers

Plant genomic DNA was isolated from fresh and young leaf tissue for each RIL and the parents following cetyltrimethyl-ammonium bromide (CTAB) method slightly modified from that used by Murray and Thompson [44]. A total of 300 simple sequence repeat (SSR) markers, RM-series [45, 46], were utilized to survey polymorphism, of which 65 polymorphic markers were well distributed among 12 chromosomes (Table 1). Polymerase chain reaction (PCR) was performed in 10 *μ*l volume containing 2 *μ*l of template DNA (25 ng), 0.4 *μ*l of forward and reverse primers each at 10 pmol concentration, 0.6 *μ*l dNTPs (2 mM), 0.12 *μ*l *Taq* DNA polymerase (5 U/*μ*l), 0.48 *μ*l MgCl_2_ (50 mM), 1 *μ*l 10X PCR buffer, and 5 *μ*l ddH_2_O. Cycling conditions followed in PCR were initial denaturation at 94°Ċ for 5 min followed by 35 cycles of denaturation at 94°Ċ for 30s, primer annealing temperature for 30s, extension at 50-58°C for 2 min and final extension at least 72°C for 5 min. After PCR amplification, the PCR products were separated on 6% polyacrylamide gels (19 : 1-acrylamide : bisacrylamide) as described by Creste et al. [47] and Bassam and Caetano-Anollés [48] and visualized by silver staining.

### 2.4. Linkage Map Construction and QTL Analysis

Out of the 300 SSR markers, 65 polymorphic SSR markers were distributed throughout 12 chromosomes of rice. Genotypic data of 157 RILs and parents were transformed according to the requirement of IciMapping software v4.0 [49] to develop the linkage map and also to identify the potential QTLs. The analysis of QTLs was carried out by using two statistical methods, namely, interval mapping (IM-ADD) and inclusive composite interval mapping (ICIM-ADD) methods [50] which were performed with a maximum recombination fraction of 0.3 and LOD threshold of 2.5 and 3.0 to claim the significant QTLs.

## 3. Results

### 3.1. Performance of Grain Appearance Traits for Parents and RIL Population

**Figure 1:**
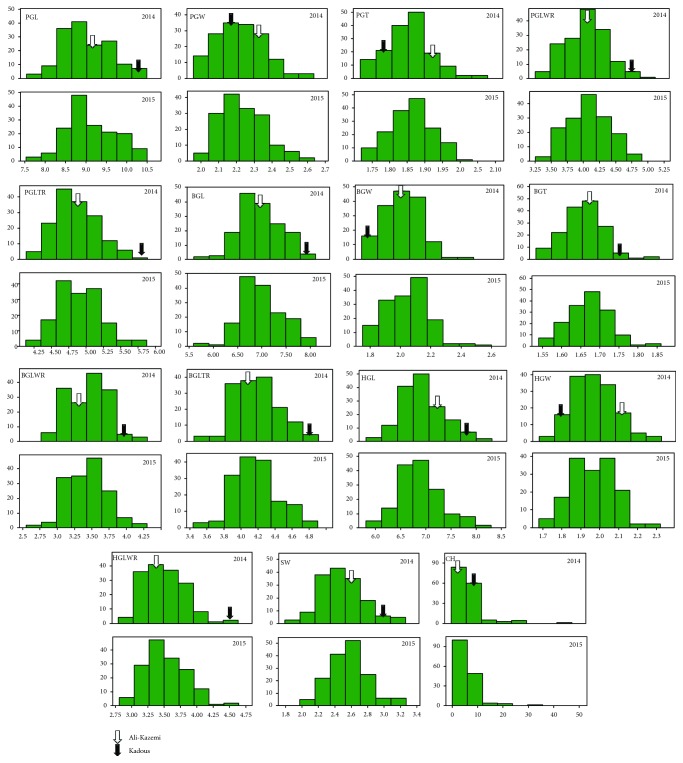
The histogram of frequency distribution of grain appearance quality traits under two seasons in rice.

**Figure 2:**
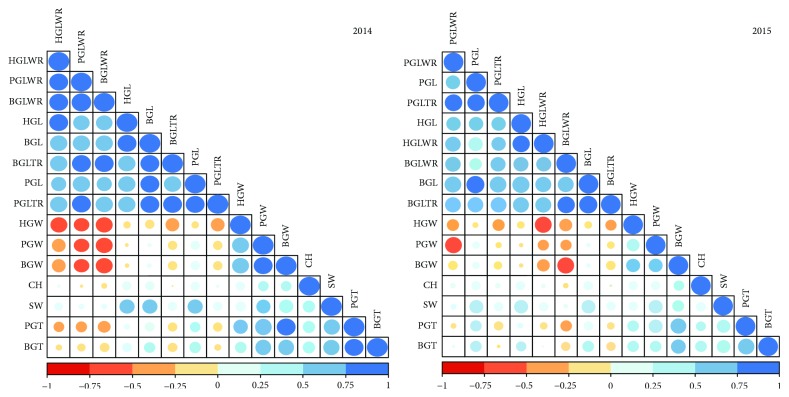
Correlation analysis of 15 traits of 157 RILs in 2014 and 2015.

The recurrent and donor parental lines (Ali-Kazemi and Kadous) and RIL population of phenotypic traits are shown in Figure 1. The average values from Kadous are higher than those from Ali-Kazemi for all 15 traits except PGL, BGW, BGT, and HGW in 2014, whereas PGW, PGT, BGW, BGT, HGW, and SW in 2015, respectively. All variables showed a positive asymmetry, varying between 0.59 (BGL) to 17.35 (CH) in 2014 and 0.05 (PGLWR) to 13.16 (CH) in 2015. Except for HGW (−0.28), the asymmetry shown was negative in 2015. The kurtosis values of four traits, PGW, BGW, BGLWR, and HGW, were negative in 2014. Similarly, in 2015, five traits, PGL, PGT, PGLWR, PGLTR, and SW, had a negative value, respectively. Therefore, the shape of the trait distribution pattern was classified as a platykurtic distribution. However, SW and CH had shown the highest coefficient variation among the RILs, whereas BGT showed the lowest CV value in both the years. Correlation analysis was carried out among the 15 traits in 2014 and 2015, and the results are represented in Figure 2. Many of the traits are positively significantly correlated with each other. The highest positive correlation was observed between PGL & PGLTR (*r* = 0.86), PGW & BGW (*r* = 0.94), PGLWR & PGLTR (*r* = 0.85), PGLWR & BGLWR (*r* = 0.91), and BGL & BGLTR (*r* = 0.88) in 2014, whereas the highest positive correlation was observed between PGL & PGLTR (*r* = 0.86) and BGL & BGLTR (*r* = 0.87) in 2015, respectively.

### 3.2. QTL Mapping for Grain Appearance Traits

**Figure 3:**
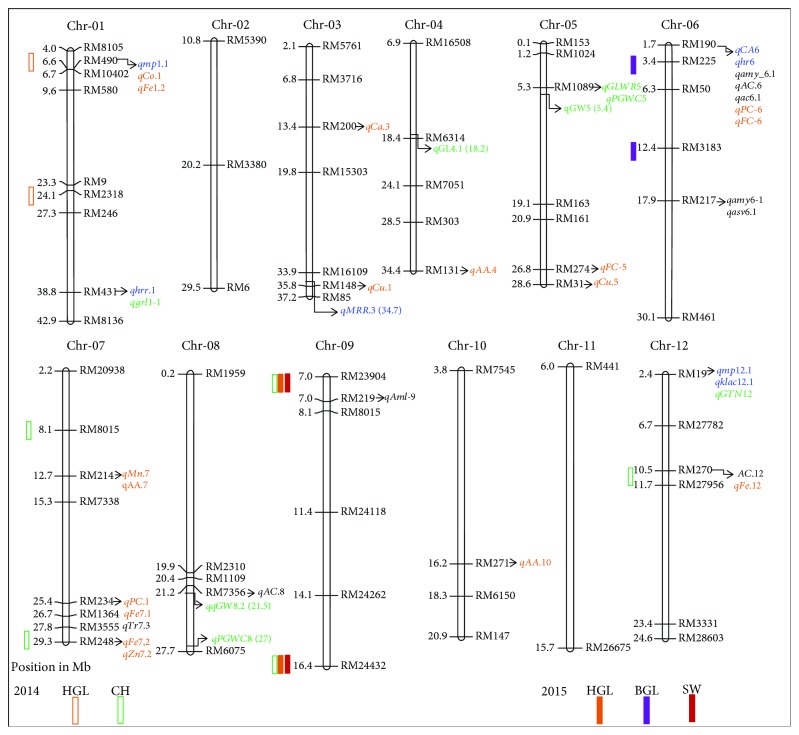
Distribution of QTLs for grain appearance and validation with previous reports on the linkage map. The hollow and solid bars indicate the QTLs detected in 2014 and 2015, respectively. The left and right sides indicate the polymorphic SSR marker physical positions (Mb) associated with previous reports. A color font of each QTL represents the classification of grain appearance and nutritional quality traits. Blue color fonts are related to milling properties of grain quality (MPGQ) QTLs, green color fonts for grain appearance (GA) QTLs, orange color fonts for nutritional factor- (NF-) related QTL (NF), and black color font for cooking property (CP) QTLs in rice.

Genetic mapping was performed by using all the 65 polymorphic SSR markers. A total of 65 RM-series markers were distributed across all chromosomes with a total coverage length of 1517.32 cM, of which chromosome 1 had the highest chromosomal spanning region of about 223.25 cM. A total of seven QTLs for grain appearance were identified on chromosomes 1, 6, 9, and 12 (Table 2; Figure 3), of which three QTLs from 2014 and four from 2015 were identified by ICIM-ADD methods. Each of these QTLs was explained by phenotypic variance (PV) which ranged from 4.02% to 10.18%, except on chromosome 6, which is associated with BGL and had shown negative additive effect of Kadous allele, whereas in the remaining, all the QTLs were contributed by the positive additive effect of Ali-Kazemi alleles.

In 2014, three QTLs (*qHGL_1*, *qCH_9*, and *qCH_12*) were distributed on chromosomes 1, 9, and 12. The QTLs were associated with two traits, HGL and CH, which explained a total PV of 16.77%, whereas in 2015, four QTLs (*qBGL_6*, *qHGL_9*, *qSW_9*, and *qSW_9-1*) were identified on chromosomes 6 and 9, respectively. The head grain length QTL *qHGL_1* is located on chromosome 1 at the 178 cM position which were flanked by RM490, and RM2318 explained a total PV of 5.01%. For grain chalkiness, two QTLs (*qCH_9*, and *qCH_12*) were detected in two chromosomes, 9 and 12, and explained a total PV of 11.76% and a LOD score of 11.05, respectively. The region flanked by RM23904 and RM24432 n chromosome 9 consisting of four QTLs, *qHGL_1*, *qHGL_9*, *qSW_9*, and *qSW_9-1*, was responsible for the head grain length and seed weight, respectively. The head grain length QTLs were noticed on chromosomes 1 and 9 in both the years 2014 and 2015. Interestingly, none of them were overlapped QTLs across both the years in the chromosomal regions. Further, we validated the identified QTLs and also polymorphic markers with previously reported QTLs for grain appearance and quality traits in rice by comprehensive literature survey and the publicly accessible Gramene database (http://archive.gramene.org).

## 4. Discussion

The physical appearance of grain dimensions (length, breadth, and length/breadth ratio) is essential for grain quality traits in rice and highly preferred by consumers. The trait of grain shape is difficult for the plant breeders to improve the grain architecture appearance by conventional breeding methods [51, 52]. The use of the marker-assisted breeding and the completion of rice genome sequencing have greatly facilitated the discovery and mapping of QTLs and genes for grain dimension. To date, several QTLs were reported for the dynamic shape of rice grain attributed traits in the various genetic background of mapping populations in rice [9, 10, 27]. However, different grain visualization traits regarding paddy, brown, and head rice grain appearance QTL information have not been reported. In this study, the focus was to dissect these traits using the RIL population, which were derived from a popular Iranian local rice cultivar of Ali-Kazemi (long-grain) and improved rice cultivar of Kadous, as well as to identify the possible DNA markers for selection of physical grain appearance traits. A total of seven QTLs were identified for four traits, HGL, BGL, SW, and CH, on the four chromosomes, 1, 6, 9, and 12, by using ICIM methods. The percentage of PV explained by each QTL was ranged from 4.02 to 10.18%, with an average of 6.32%. Among these seven QTLs, single QTL *qSW_9-1 (2015)* which accounted for PV was higher than 10%, while the remaining six QTLs explained ranged from 4.02 to 7.22% (Table 2). The higher phenotypic variation explained for most of the studied traits revealed major genes/QTLs to be responsible. Out of the 15 traits, four traits were found to be governed by one or two QTLs that explained a significant PV. However, only QTLs for a single-trait HGL flanked by RM490-RM2318 on chromosome 1 and RM23904 and RM24432 on chromosome 9 were identified in both the years 2014 and 2015. These QTLs are contributed by the positive additive effect of Ali-Kazemi allele.

### 4.1. Validation of the Identified QTLs and Polymorphic Markers and Their Significance

To date, several researchers had identified QTLs that were associated with physical appearance and grain quality traits and were located majorly on chromosomes 1, 3, 4, 5, 6, 7, and 10 [5, 10, 27, 36, 39]. In the present study, paddy, brown, and head grain rice appearance trait-related QTLs and polymorphic SSR markers were effectively compared with previously reported QTLs and linkages maps. The QTL intervals of RM490 to RM2318 on chromosome 1, RM225 to RM3183 on chromosome 6, RM23904 to RM24432 on chromosome 9, RM270 to RM27956 on chromosome 12, and also a few polymorphic SSR markers were associated with four different key component traits as follows: milling properties of grain quality (MPGQ), grain appearance (GA), nutritional factors (NF), and cooking properties (CP) in rice.

In our study, on chromosome 1, a single QTL *qHGL_1 (2014)* flanked by right SSR marker RM490 was significantly associated with three QTLs *qmp1.1* [53], *qCo.1* [36], and *qFe1.2* [54]. Also, we found that these QTLs were responsible for milling and mineral element content related to nutritional factors in grain quality. Interestingly, the polymorphic RM431 marker used in our study was associated with two QTLs related to head rice recovery (*qhrr.1*) and grain length (*qgrl1-1). qhrr.1* identified by using BC_2_F_2_ population was derived from a genetic background of IR64 and *O. rufipogon* mapping populations [55], whereas *qgrl1-1* was reported by Amarawathi et al. [5] by using RILs from Pusa 1121 and Pusa 1342. Likewise, adjacent to the polymorphic marker RM8136 at the 42.7 Mb region, *qBRR1.2* (brown rice rate) QTL was associated that accounted for 7.1% of PV [10].

Lu et al. [56] identified a total of 10 QTLs for five different mineral elements such as Cu, Ca, Zn, Mn, and Fe on six chromosomes 1, 2, 4, 5, 7, and 9, which explained a PV ranging from 5.3 to 25.81%. Among these, two mineral element QTLs, *qCa.3* and *qCu.1*, were similarly mapped on chromosome 3 which were associated with polymorphic marker RM200 used in our study at 13.4 Mb and RM148 at 35.8 Mb regions. Wang et al. [10] further detected that for the brown rice rate QTL, *qBRR3* was adjacent to RM16109 at 34.7 Mb that accounted for 6.9% of PV. Similarly, RM131 at 34.4 Mb and the adjacent RM6314 at the 18.2 Mb region on chromosome 4 were controlling the amino acid content in rice grain (*qAA.1*) [57] and grain length (*qGL4.1*) [10]. The polymorphic marker used in our study has been found to be tightly linked to several important grain quality and nutrient traits. Such polymorphic markers could be fully utilized in rice breeding programs to mobilize the grain quality traits of interest in early generations.

Recently, Wang et al. [10] identified 72 QTLs for nine rice grain appearances, and milling quality traits were distributed on 12 chromosomes in two different environments by using a diverse panel of 258 rice accessions from the 3K Rice Genome Project. Out of 72, 11 QTLs were overlapped or adjacent to the SSR marker positions on six different chromosomes, 1, 3, 4, 5, 7, and 8, in the current study. On chromosome 5, two QTLs *qGLWR5* and *qPGWC5* associated with RM1089 at 5.3 Mb regions and *qGW5* adjacent 5.4 Mb regions responsible for grain length to width ratio (GLWR), the percentage of grain with chalkiness (PGWC) and grain width (GW), respectively. Two QTLs for fat content (*qFC-5*, Yu et al. [58]) and microelement (*qCu.5*, Lu et al. [56]) were associated with RM274 and RM31. Polymorphic markers to select for fat content and microelements can be highly useful for fair-sized rice breeding programs.

On chromosome 6, single QTL *qBGL_6 (2015)* were mapped at 52 cM regions was flanked by RM225 and RM3183, which is associated with BGL, and it was explained by a PV of 6.70%. This QTL was identified as a novel QTL and has not been previously reported. Near the RM225, polymorphic RM190 located at the 1.7 Mb region which was significantly associated with seven QTLs, namely, chalkiness-*qCA6* [59], head rice recovery-*qhr6* [14, 33], protein and fat content-*qPC_6* and *qFC_6* [58], and three amylose content QTLs-*qamy_6.1*, *qAC.*6, and *qac6.1* [14, 60, 61] were reported. These QTLs were identified with the genetic background of RILs derived from a cross between Xieqingzao B and Milyang [58], ZS97 and NYZ [59], Cypress and Panda [14], and another population of DHs derived from Caiapo and *O. glaberrima* [33]. Polymorphic markers like RM190 hold a high value and could be used directly in the marker-assisted selection of multiple traits.

The polymorphic markers RM214 (12.7 Mb) associated with *qMn.7* [36] and *qAA.7* [57]; RM234 (25.3 Mb) for three QTLs, *qPC.1* [62], *qFe7.1* [54], and *qTr7.3* [10]; and also adjacent to RM8015 (8.4 Mb) for single QTL *qBRR7* [10] were responsible for various milling properties of grain quality and nutritional mineral element contents in rice grain. Four QTLs for three traits, CH, HGL, and SW, were detected by ICIM. These include one QTL *qCH_1* in 2014 and the remaining three QTLs *qHGL_9*, *qSW_9*, and *qSW_9-1* were identified in 2015, which explained 5.01%, 7.22%, 6.08%, and 10.18%, respectively. The clusters of QTLs were not reported by previous results of grain appearance and nutritional quality trait-related QTLs and markers. On chromosome 12, single QTL *qCH_12* for CH was identified by the ICIM method.

To date, more than 82 QTLs governing chalkiness-related traits (http://archive.gramene.org/qtl/) including 30 QTLs for percentage grain with white core (PGWC), 26 QTLs for degree of endosperm chalkiness (DEC), 12 QTLs for area of endosperm chalkiness (AEC), 11 QTLs for white-backed kernel, and 3 QTLs for basal white (BW) were reported [28, 63]. However, recently, two significant QTLs for chalkiness related as PGWC (*qPGWC6*) and chalkiness score (CS) (*qCS6*) on chromosome 6 were reported by Chandusingh et al. [64]; 79 QTLs for six chalkiness-related traits as white core rate, white core area, white belly rate, white belly area chalkiness rate, and chalkiness area on 12 chromosomes by Peng et al. [59]; and also 19 QTLs for chalkiness on six different chromosomes, 1, 4, 6, 7, 9, and 12, by Gao et al. [65] reported by using DHs and RIL populations. By comparison of previously reported QTLs and databases, *qCH_12* flanked by right marker RM270 was associated with three nutritional-related QTLs such as *qAC.12* [60] and *qFe.12* [66] by using DH populations (Yuefu/IRAT109 and IR64/Azucena). The polymorphic markers RM219 on chromosome 9 (*qAml-9*), RM271 on chromosome 10 (*qAA.10*), and RM19 on chromosome 12 (*qmp12.1*, *qklac12.1*, and *qGTN12*) were associated with MPGQ-, NF-, and GA-related traits [53, 57, 67]. In this study, we have validated the identified QTLs and polymorphic SSR markers based on published resources and databases. Several polymorphic markers and identified QTLs were associated with more than two traits and colocalized on chromosomes 1, 6, and 9, which could be utilized to improve the selection efficiency in rice breeding programs. The multiple traits on single locus may have multiple effects on each other as they belong to the identical genomic position. These could be due to a pleiotropic effect of a single gene or certain genes coexisting in the same QTLs. A further deeper understanding of fine mapping of the target genomic region would provide a genetic inheritance pattern and better picture to understand the linkage or pleiotropic effects on grain appearance and quality trait in rice.

## 5. Conclusion

Grain appearance and nutritional quality traits are vital for commercial rice production, and they also affect the dietary value of the grain. Considerable grain appearance traits in paddy, brown, and head rice were observed in the current study. Through ICIM-ADD methods, a total of seven QTLs for four traits, namely, HGL, BGL, SW, and CH, were identified on four chromosomes, 1, 6, 9, and 12. By comparison of the comprehensive literature survey and publically accessible Gramene database, 19 polymorphic markers were significantly associated with several traits related to MP, GA, CP, and NF. The four trait-associated QTLs are novel on chromosomes 6 and 9. Therefore, these QTLs and validated SSR polymorphic markers could provide valuable information for multiple traits related to grain appearance and nutritional quality for the future marker-assisted breeding program for improving desirable traits in rice.

## Figures and Tables

**Table 1:** List of polymorphic rice microsatellite (RM) markers used in the QTL mapping.

S. no.	Markers	Chr	Mb	Repeat motif	Tm	Exp. size	Forward primer	Reverse primer
1	RM 8105	1	4	(AG)18	55	100	TCATTCTCGAAGGCTTACGG	TCAAGCCTAAGCAGAGGATG
2	RM 580	1	9.6	(CTT)19	55	221	GATGAACTCGAATTTGCATCC	CACTCCCATGTTTGGCTCC
3	RM 490	1	6.6	(CT)13	55	101	ATCTGCACACTGCAAACACC	AGCAAGCAGTGCTTTCAGAG
4	RM 9	1	23.3	(GA)15GT(GA)2	55	136	GGTGCCATTGTCGTCCTC	ACGGCCCTCATCACCTTC
5	RM 10402	1	6.69	(CT)19	55	286	TGGATTGAAGGGAGCTCTACACC	TTGCTCCACACGATCTACACAGC
6	RM 2318	1	24.1	(AT)25	55	133	CTTTTGCTCATCCATTCG	CCTCTTCATGCGATAAACAT
7	RM 8136	1	42.9	(AT)71	55	225	ATGTAAGCTAGGTAGAGCTG	GCGTACGTACGTAAGTAATA
8	RM 246	1	27.3	(CT)20	55	116	GAGCTCCATCAGCCATTCAG	CTGAGTGCTGCTGCGACT
9	RM 431	1	38.8	(AG)16	55	251	TCCTGCGAACTGAAGAGTTG	AGAGCAAAACCCTGGTTCAC
10	RM 5390	2	10.8	(CA)23	55	107	CTCGACCAAACAGACCAGTAGGG	ATCGCCGCTTAGGAGAATCTGG
11	RM 6	2	29.5	(AG)16	55	163	GTCCCCTCCACCCAATTC	TCGTCTACTGTTGGCTGCAC
12	RM 13380	2	20.2	(TC)10	55	177	GCCTTTCTCTTGATCTCCTCGATCC	CTGGACACTGCGGTTGCTTCC
13	RM 85	3	37.2	(TGG)5(TCT)12	55	107	CCAAAGATGAAACCTGGATTG	GCACAAGGTGAGCAGTCC
14	RM 16109	3	33.9	(TC)16	55	292	CAGAAACTGAGGAGAGAGAAAGATCG	CGATTCAGAGCCATGCTACCG
15	RM 3716	3	6.83	(AG)17	55	127	GTCGTTCGGTTGACTCGTTG	CACACATATATACCCCCCCC
16	RM 148	3	35.8	(TG)12	55	129	ATACAACATTAGGGATGAGGCTGG	TCCTTAAAGGTGGTGCAATGCGAG
17	RM 200	3	13.4	(GA)16	55	122	CGCTAGGGAATTTGGATTGA	CGATGAGCAGGTATCGATGAGAAG
18	RM 15303	3	19.8	(TCT)15	55	199	GAATCGGGTCTACGGTTTAGG	AAAGGAAGAGAAGAGGCAACG
19	RM 5761	3	2.1	(AGA)8	55	104	AGGGGAATGGCAAGATTACC	AGTCGTCCTCTTCACATGGC
20	RM 6314	4	18.4	(CTT)11	50	169	GATTCGTGTCGGTTGTCAAG	GGTTCAGGGACGAATTTCAG
21	RM 7051	4	24.1	(AATC)7	55	176	CTCGATGAGCTTGGCGTC	TTCAGTGTTCATCGCCTCTG
22	RM 16508	4	6.9	(GCC)7	55	188	TTCATTGTCATCGCCTCATTGG	ACAGGTACAGCTGGGTAGAGAGAAGC
23	RM 131	4	34.4	(CT)9	61	215	TCCTCCCTCCCTTCGCCCACTG	CGATGTTCGCCATGGCTGCTCC
24	RM 303	4	28.5	[AC(AT)2-10]9(GT)7(ATGT)6	55	200	GCATGGCCAAATATTAAAGG	GGTTGGAAATAGAAGTTCGGT
25	RM 1024	5	1.2	(AC)13	55	141	GCATATACCATGGGGATTGG	GGGATTGGGATAATGGTGTG
26	RM 1089	5	5.3	(AC)33	55	239	CAGAAGGATTATCTCGATACC	AATAGGGCTTGAAATAAATTG
27	RM 161	5	20.9	(AG)20	61	187	TGCAGATGAGAAGCGGCGCCTC	TGTGTCATCAGACGGCGCTCCG
28	RM 163	5	19.1	(GGAGA)4(GA)11C(GA)20	55	124	ATCCATGTGCGCCTTTATGAGGA	CGCTACCTCCTTCACTTACTAGT
29	RM 274	5	26.8	(GA)15-7-(CGG)5	55	160	CCTCGCTTATGAGAGCTTCG	CTTCTCCATCACTCCCATGG
30	RM 31	5	28.6	(GA)15	55	140	GATCACGATCCACTGGAGCT	AAGTCCATTACTCTCCTCCC
31	RM 190	6	1.7	(CT)11	55	124	CTCTCTCACCATTCCTTCAGTTC	GCAAGACTGGTTTCCACATGG
32	RM 225	6	3.4	(CT)18	55	140	TGCCCATATGGTCTGGATG	GAAAGTGGATCAGGAAGGC
33	RM 3183	6	12.4	(CT)12	50	140	GCTCCACAGAAAAGCAAAGC	TGCAACAGTAGCTGTAGCCG
34	RM 461	6	30.1	(AAAC)6	55	195	GAGACCGGAGAGACAACTGC	TGATGCGGTTTGACTGCTAC
35	RM 217	6	17.9	(CT)20	55	133	ATCGCAGCAATGCCTCGT	GGGTGTGAACAAAGACAC
36	RM 50	6	6.3	(CTAT)4(CT)15	55	201	ACTGTACCGGTCGAAGACG	AAATTCCACGTCAGCCTCC
37	RM 8015	7	8.1	(AT)34	55	130	AAGTTTCTCCAAGCCAAGAG	AATGTGTTTTCCTGGTCAGA
38	RM 1364	7	26.7	(AG)26	55	158	AAGAAATTCAAAACACATGA	AAAACATCTACTTTGATCCA
39	RM 3555	7	27.8	(GA)12	55	154	TGGAAGTTTCCTGGCGATAG	TGGTTGGACTGAAAAGTCCC
40	RM 7338	7	15.3	(CATC)9	55	164	CTTATCTCTCGGCAAGCAGC	CTCACACGCATGGATCAATC
41	RM 214	7	12.7	(CT)14	55	112	CTGATGATAGAAACCTCTTCTC	AAGAACAGCTGACTTCACAA
42	RM 234	7	25.4	(CT)25	55	156	ACAGTATCCAAGGCCCTGG	CACGTGAGACAAAGACGGAG
43	RM 248	7	29.3	(CT)25	55	102	TCCTTGTGAAATCTGGTCCC	GTAGCCTAGCATGGTGCATG
44	RM 20938	7	2.2	(GAG)8	57	454	GGACATCTACTCGCAGCTCTGG	TTACACGCTCTGACAGGTTGTGG
45	RM 2310	8	19.9	(GA)10	55	295	CGAGTAGCGGAACTGGATGAACTCC	GTTCAGGGTACGCGCCAAACG
46	RM 1959	8	0.2	(AT)19	55	160	CTATTGTACCTGCTCTCATC	ACATCGGTACTGATAATGTT
47	RM 1109	8	20.4	(AG)12	55	198	TCAAAATCACGTGTATGTAAGC	TTTACAAAGGACAGAGGGC
48	RM 24432	9	16.4	(TA)29	50	638	GTGTGAGTTTGGTTTGGGAGAGG	GCCCAATACACGGTAGATTCATCC
49	RM 219	9	7.8	(CT)17	55	202	CGTCGGATGATGTAAAGCCT	CATATCGGCATTCGCCTG
50	RM 23904	9	7	(CT)11	55	164	CTCACCGGAGCACCACTAACC	GAGAGCAAGACTGTGAAGTGTGAACC
51	RM24118	9	11.1	(TA)11	55	398	TCTCAATAGTCGCCACCAACACC	GAGCCCGGCAGAAATTTAAAGC
52	RM24262	9	14.1	(ACAT)5	55	255	CTCATCGGCGACATATCACAGC	CTCATCGGCGACATATCACAGC
53	RM8015	9	8.1	(AT)34	55	130	AAGTTTCTCCAAGCCAAGAG	AATGTGTTTTCCTGGTCAGA
54	RM 7545	10	3.8	(TATG)18	61	225	GTATCCGCTCCGTTTTCATC	GAGGGGGGGGTGTAGAATAG
55	RM 6150	10	18.3	(CGC)9	50	180	CTCGACGGAGCTCTCTTCAC	CAAGAAGCAGAGGAAAAGCG
56	RM 147	10	20.9	(TTCC)5(GGT)5	55	97	TACGGCTTCGGCGGCTGATTCC	CCCCCGAATCCCATCGAAACCC
57	RM 271	10	16.2	(GA)15	55	101	TCAGATCTACAATTCCATCC	TCGGTGAGACCTAGAGAGCC
58	RM 441	11	6	(AG)13	55	189	ACACCAGAGAGAGAGAGAGAGAG	TCTGCAACGGCTGATAGATG
59	RM 26675	11	15.7	(ACG)8	55	262	AGCACAGTGTTCACCAGCATTGG	ACGTGGTCGACGAAGGTGACG
60	RM 270	12	10.5	(GA)13	55	108	GGCCGTTGGTTCTAAAATC	TGCGCAGTATCATCGGCGAG
61	RM 19	12	2.4	(ATC)10	55	226	CAAAAACAGAGCAGATGAC	CTCAAGATGGACGCCAAGA
62	RM 27956	12	11.7	(CT)10	55	173	AGCAACCTACCTTGCCAAATTACC	TGTGCATATCCATTGACACAGC
63	RM 3331	12	23.4	(CT)15	50	129	CCTCCTCCATGAGCTAATGC	AGGAGGAGCGGATTTCTCTC
64	RM 27782	12	6.7	(ATT)13	55	414	GAGGAGAGGAGACGGAGAGG	CGAGAGTGGTGATCTCACTTAATAGG
65	RM 28603	12	24.6	(CT)12	55	392	ATCCCGACCTAGGATACGGTTGC	CATGGAGTGTGAGTTCCAAATTGC

**Table 2:** Additive effects of QTLs for grain appearance detected by ICIM-ADD in two years.

S. no.	QTL	Chr	Position (cM)	Flanking markers	LOD^a^	PVE^b^	Additive effect^c^	The contribution of the favorable allele
1	*qHGL_1* (2014)	1	178	RM490-RM2318	10.26	5.01	10.57	A
2	*qCH_9* (2014)	9	77	RM23904-RM24432	2.95	6.7	0.23	A
3	*qCH_12* (2014)	12	54	RM270-RM27956	8.1	5.06	10.25	A
4	*qBGL_6* (2015)	6	52	RM225-RM3183	3.01	4.02	-0.07	K
5	*qHGL_9* (2015)	9	77	RM23904-RM24432	2.86	7.22	0.23	A
6	*qSW_9* (2015)	9	78	RM23904-RM24432	2.57	6.08	0.21	A
7	*qSW_9-1* (2015)	9	82	RM23904-RM24432	3.02	10.18	0.11	A

^a^Interval of the LOD peak value for QTL; ^b^interval of the LOD peak value for QTL; ^c^positive additive effect of QTL came from Ali-Kazemi, while negative effect came from Kadous.

## Data Availability

All the relevant data has been incorporated into the original research manuscript.

## Supplementary Materials

Supplementary Materials Supplementary Table 1: list of rice grain appearance and quality traits of associated QTLs in the different genetic background of mapping populations. Supplementary Table 2: a list of SSR markers has been used in the QTL mapping study.
