# Supplementary material for: Genetic Mapping and Validation of Quantitative Trait Loci (QTL) for the Grain Appearance and Quality Traits in Rice (Oryza sativa L.) by Using Recombinant Inbred Line (RIL) Population
Source: Int J Genomics. 2019 Feb 25;2019:3160275. doi: 10.1155/2019/3160275 (PMC6410440; doi:10.1155/2019/3160275)
Supplement: Supplementary Materials — Supplementary Table 1: list of rice grain appearance and quality traits of associated QTLs in the different genetic background of mapping populations. Supplementary Table 2: a list of SSR markers has been used in the QTL mapping study. [file 3160275.f1.docx]

**Supplementary Table 1 List of rice grain appearance and nutrient quality traits associated with different QTLs (>3.0 LOD) mapped in different rice population**

| **S. No** | **Grain traits** | **Chr** | **QTLs** | **Markers** | **Type** | **Peak marker** | | **Populations** | **References** |
| --- | --- | --- | --- | --- | --- | --- | --- | --- | --- |
| **A** | **MPGQ- Milling properties of grain quality** | | | | | | | | |
| 1 | BRR | 1 | *qbr1* | RM297-RM315 | DHs |  | | Caiapo/*O.glaberrima* | [1] |
| 2 | KWB | 1 | *qkwb1* | E23M61.230 | RILs |  | | Cypress/Panda | [2] |
| 3 | HRR | 1 | *qhrr.1* | RM431 | BC_2_F_2_ | RM431 | | IR64/*O. rufipogon* | [3] |
| 4 | HRR | 1 | *qhr.1* | SS3994-RM8137 | CSSLs |  | | Koshihikari/Nona Bokra | [4] |
| 5 | BRR | 1 | *qbr_1.1* | RM5638-RM1361 | RILs |  | | L204/01Y110 | [5] |
| 6 | MRR | 1 | *qmr_1.1* | RM5638-RM1361 | RILs | RM5638 | |  |  |
| 7 | MP | 1 | *qmp1.1* | RM490-RM243 | BC_2_F_2_ |  | | Swarna/IRGC81848 | [6] |
| 8 | KL | 1 | *qkl1.1* | RM499-RM84 | BC_2_F_2_ |  | |  |  |
| 9 | MRR | 2 | *qmr2* | E13M47.411 | RILs |  | | Cypress/Panda | [2] |
| 10 | RB | 2 | *qrb2* | RM236-RM279 | DHs | RM236-RM279 | | Caiapo/*O.glaberrima* | [1] |
| 12 | KW | 2 | *qkw_2.1* | RM6911-RM12987 | RILs |  | | L-202/Lemont | [7] |
| 13 | HRR | 2 | *qHRR-2* | RM3874-RM5651 | RILs |  | | Chuan7/Nanyanghan | [8] |
| 14 | MP | 3 | *qmp3.1* | RM7-RM16 | BC_2_F_2_ |  | | Swarna/IRGC81848 | [6] |
| 15 | KL | 3 | *qkl_3.1* | RM569-RM3117 | RILs |  | | L-202/Lemont | [7] |
| 16 | HRR | 3 | *qHr3* | RM81B-RM7 | DHs |  | | Caiapo/*O.glaberrima* | [1] |
| 17 | KW | 3 | *qkwt_3.1* | RM5626-RM6736 | RILs |  | | L-202/Lemont | [7] |
| 18 | BRR | 3 | *qBRR-3* | RM3204-RM6283 | RILs | RM3204-RM6283 | | Chuan7/Nanyanghan | [8] |
| 19 | MHP | 3 | *qMHP-3* | G1316 | RILs |  | | Asominori/IR24 | [9] |
| 20 | BRR | 3 | *qbr3* | RM5474 | RILs |  | | Cypress/Panda | [2] |
| 21 | KLB | 4 | *qklb4* | E23M47.072 | RILs |  | |  |  |
| 22 | KLH | 4 | *qklh4* | E23M47.072 | RILs |  | |  |  |
| 23 | MP | 4 | *qmp4.1* | RM261-RM241 | BC_2_F_2_ | RM261-RM241 | | Swarna/IRGC81848 | [6] |
| 24 | VER | 5 | *qver5.1* | RM430-RM26 | BC_2_F_2_ |  | |  |  |
| 25 | HRR | 5 | *qhrr.5* | RM178 | BC_2_F_2_ | RM178 | | IR64/*O. rufipogon* | [3] |
| 26 | BRR | 5 | *qbr.5* | RM42-C734b | RILs |  | | Zhenshan97/Minghui 63 | [10] |
| 27 | CA | 6 | *qCA6* | RM276-RM549 | RILs |  | | ZS97/DL208 | [11] |
| 28 | CR | 6 | *qCR6* | RM527-RG2498 | RILs |  | | ZS97/NYZ |  |
| 29 | CA | 6 | *qCA6* | RM190-RM587 | RILs |  | |  |  |
| 30 | CS | 6 | *qCS6* | RM6273-RM204 | RILs | RM6273-RM204 | | Pusa1266/Jaya | [12] |
| 31 | PGWC | 6 | *qPGWC6* | RM6273-RM204 | RILs |  | |  |  |
| 32 | HRR | 6 | *qhr6* | RM190 | RILs |  | | Cypress/Panda | [2] |
| 33 | KW | 6 | *qkw_6.1* | RM3353 | RILs |  | | L204/01Y110 | [5] |
| 33 | HR | 6 | *qhr6* | RM190-RM253 | DHs |  | | Caiapo/*O.glaberrima* | [1] |
| 34 | HRR | 6 | *qhr6* | RM190-RM253 | DHs |  | |  |  |
| 35 | HRR | 6 | *qhr_6.1* | RM3414-RM3567 | RILs |  | | L204/01Y110 | [5] |
| 36 | CR | 6 | *qCR6* | RM435-RM170 | DHs | RM435-RM170 | | ZS97/H94 | [11] |
| 37 | BRR | 7 | *qBr7* | RM10-RM351 | DHs |  | | Caiapo/*O.glaberrima* | [1] |
| 38 | MRR | 7 | *qmr7* | RM125-RM11 | DHs |  | |  |  |
| 39 | HRR | 8 | *qhr.8* | RM5911-SA1656 | CSSLs |  | | Koshihikari/Nona Bokra | [4] |
| 40 | CA | 9 | *qCA9* | RM278-RM553 | DHs |  | | ZS97/H94 | [11] |
| 41 | CR | 9 | *qCR9* | RM159-RM524 | RILs |  | | ZS97/DL208 |  |
| 42 | MRR | 9 | *qmr_9.1* | RM8206-RM23998 | RILs |  | | L204/01Y110 | [5] |
| 43 | HRR | 9 | *qhr_9.1* | RM566-RM1189 | RILs |  | | L-202/Lemont | [7] |
| 44 | KTN | 9 | *qkthk_9.1* | RM2420-RM566 | RILs |  | |  |  |
| 45 | BRP | 9 | *qBRP-9* | XNpb108 | RILs |  | | Asominori/IR24 | [9] |
| 46 | KL | 10 | *qkl_10.1* | RM25766 | RILs |  | | L204/01Y110 | [5] |
| 47 | KW | 10 | *qkw_10.1* | RM25766-RM25871 | RILs | RM25871 | |  |  |
| 48 | KTN | 10 | *qkthk_10.1* | RM25766 | RILs |  | |  |  |
| 49 | MRR | 10 | *qmrr.10* | RRM474 | BC_2_F_2_ | RM474 | | IR64/*O. rufipogon* | [3] |
| 54 | RB | 10 | *qrb10* | RM184-RM171 | DHs |  | | Caiapo/*O.glaberrima* | [1] |
| 55 | BRP | 10 | *qBRP-10* | C1166 | RILs |  | | Asominori/IR24 | [9] |
| 56 | MRP | 11 | *qMRP-11* | XNpb257 | RILs |  | |  |  |
| 57 | HRR | 11 | *qhr_11.1* | RM26073-RM167 | RILs |  | | L204/01Y110 | [5] |
| 58 | MP | 12 | *mp12.1* | RM415-RM19 | BC_2_F_2_ | RM415-RM19 | | Swarna/IRGC81848 | [6] |
| 59 | KLC | 12 | *klac12.1* | RM19-RM453 | BC_2_F_2_ |  | |  |  |
| **B** | **GA- Grain appearance** | | | | | | | | |
| 60 | GL | 1 | *qGL-1* | RM579-RM23 | ILs |  | | Teqing/lemont | [13] |
| 61 | GL | 2 | qGL2-1 | RM29-RM262 | RILs |  | | Asominori/IR24 | [14] |
| 62 | GL | 2 | qGL2-2 | RM6-RM425 | RILs |  | |  |  |
| 63 | GL | 3 | qGL3-1 | RM411-RM7097 | RILs |  | |  |  |
| 64 | GL | 7 | qGL7 | RM505-RM234 | RILs |  | |  |  |
| 65 | GL | 8 | qGL8-1 | RM6863-RM72 | RILs |  | |  |  |
| 66 | GL | 8 | qGL8-2 | RM502-RM6948 | RILs |  | |  |  |
| 67 | GW | 3 | qGW3-1 | RM411-RM7097 | RILs |  | |  |  |
| 68 | GW | 5 | qGW5 | RM267-RM405 | RILs |  | |  |  |
| 69 | LW | 3 | qLW3 | RM411-RM7097 | RILs |  | |  |  |
| 70 | LW | 5 | qLW5 | RM267-RM405 | RILs |  | |  |  |
| 71 | GCF | 2 | qGCF2-2 | RM6-RM425 | RILs |  | |  |  |
| 72 | GCF | 3 | qGCF3-1 3 | RM411-RM7097 | RILs |  | |  |  |
| 73 | GCF | 8 | qGCF8 | RM502-RM6948 | RILs |  | |  |  |
| 74 | GL | 1 | *qgl.1* | RM104 | LT/TL-RILs |  | | Lemont/TeQing | [15] |
| 75 | GL | 1 | *qgl.1* | RM156 | LT/TL-RILs |  | |  |  |
| 76 | GL | 1 | *qgrl1-1* | RM431-RM104 | RILs | RM431-RM104 | | Pusa1121/Pusa1342 | [16] |
| 77 | GL | 1 | *qGRL-1.1* | RM226-RM104 | RILs |  | |  |  |
| 78 | GW | 1 | *qGW-1* | R1613-XNpb216 | RILs |  | | Asominori/IR24 | [9] |
| 79 | GL | 2 | *qGl.2* | RM438-RM341 | F_2_ |  | | Domsephid/Gerdeh | [17] |
| 80 | GB | 2 | *qGb.2* | RM279-RM555 | F_2_ |  | |  |  |
| 81 | GS | 2 | *qgs.2* | RM475-RM263 | F_2_ |  | |  |  |
| 83 | GL | 2 | *qGL2.2* | RM341-RM262 | RILs |  | | TD70/Kasalath | [18] |
| 84 | GT | 2 | *qGT2.1* | RM109-RM3264 | RILs |  | |  |  |
| 85 | GW | 2 | *qGW2.2* | RM1347-RM5699 | RILs | RM1347-RM5699 | |  |  |
| 86 | GW | 2 | qGW2 | W004 | F2 |  | | WY3/Fengaizhan-1 | [19] |
| 87 | GW | 2 | *qGW2.2* | RM1347-RM5699 | RILs |  | | TD70/Kasalath | [18] |
| 88 | GTN | 2 | *qGTN2.3* | RM1347-RM5699 | RILs |  | |  |  |
| 89 | GL | 2 | *qGL-2* | RM452-RM324 | ILs |  | | Teqing/lemont | [13] |
| 90 | GE | 2 | *qGE-2-1* | RM53-RM174 | F_2_ |  | | Basmati370/MR84 | [20] |
| 91 | GW | 2 | *qGW-2b* | RM3874-RM5651 | RILs |  | | Chuan7/Nanyanghan | [8] |
| 92 | GL | 3 | *qGL-3* | RM6283-RM16 | RILs | RM6283-RM16 | |  |  |
| 93 | GL | 3 | *qGL.3* | RM251-RM554 | F_2_ | RM251-RM554 | | Domsephid/Gerdeh | [17] |
| 94 | GB | 3 | *qGB.3* | RM7-RM251 | F_2_ | RM7-RM251 | |  |  |
| 95 | GS | 3 | *qGS.3* | RM251-RM554 | F_2_ | RM251-RM554 | |  |  |
| 96 | GL | 3 | *qGL3.1* | RM6080-RM6832 | RILs | RM6080-RM6832 | | TD70/Kasalath | [18] |
| 97 | GTN | 3 | *qGTN3.1* | RM6080-RM6832 | RILs |  | |  |  |
| 98 | GL | 3 | *qgl3.1* | RG393-C1087 | F_2:3_ |  | | Zhenshan97/Minghui63 | [21] |
| 99 | LBR | 3 | *qGW3.1* | RG393-C1087 | RILs |  | |  |  |
| 100 | GW | 4 | *qGW-4* | C335-C6212 | RILs |  | | Asominori/IR24 | [22] |
| 101 | GV | 4 | *qGV.4* | RM348 | LT-RILs |  | | Lemont/TeQing | [15] |
| 102 | GL | 4 | *qGL-4* | RM119-RM252 | ILs |  | | Teqing/lemont | [13] |
| 103 | GS | 5 | *qGS.5* | RM437- RM289 | F_2_ |  | | Domsephid/Gerdeh | [17] |
| 104 | GB | 5 | *qGB.5* | RM437- RM289 | F_2_ |  | |  |  |
| 105 | GTN | 5 | *qGTN.5* | RM13 | LT-RILs |  | | Lemont/TeQing | [15] |
| 106 | GW | 5 | *qGW5.2* | RM169-RM1237 | RILs |  | | TD70/Kasalath | [18] |
| 107 | GW | 5 | qSW5 | MS40671 | F2 |  | | Nipponbare/Kasalath | [23] |
| 108 | GW | 5 | *qGW.5* | RM437 | LT/TL-RILs |  | | Lemont/TeQing | [15] |
| 109 | GW | 5 | *qGW5.1* | RG360-C734a | F_2:3_/ RILs |  | | Zhenshan97/Minghui 63 | [21] |
| 110 | GW | 5 | *qGW-5* | Y1060L-R569 | RILs |  | | Asominori/IR24 | [22] |
| 111 | GB | 6 | *qGB.6* | RM527-RM3 | F_2_ |  | | Domsephid/Gerdeh | [17] |
| 112 | GV | 6 | *qGV.6* | RM19691 | TL-RILs |  | | TeQing/Lemont | [15] |
| 113 | GL | 7 | *qGRL-7.1* | RM11-RM505 | RILs | RM11-RM505 | | Pusa1121/Pusa1342 | [24] |
| 114 | LBR | 7 | *qlbr7-1* | RM505-RM336 | RILs |  | | Pusa1121/Pusa1342 | [5] |
| 115 | GB | 7 | *qgrb7-1* | RM11-RM505 | RILs |  | |  |  |
| 116 | GW | 7 | *qGW.7* | RM478 | LT/TL-RILs |  | | Lemont/TeQing | [15] |
| 117 | GL | 7 | *qGL.7* | RM478 | LT/TL-RILs |  | | Lemont/TeQing | [15] |
| 118 | GL | 7 | *qGL7.2* | RM1209-RM3264 | RILs |  | | TD70/Kasalath | [18] |
| 119 | GL | 7 | *qgrl 7.1* | RM505 | RILs |  | | Pusa Basamati1121/Pusa1342 | [16] |
| 120 | GL | 7 | *qGL.7* | RM481-RM125 | F_2_ |  | | Domsephid/Gerdeh | [17] |
| 121 | GL | 8 | *qGL.8* | RM72-RM515 | F_2_ |  | |  |  |
| 122 | GB | 8 | *qGB.8* | RM256-RM230 | F_2_ |  | |  |  |
| 125 | GS | 8 | *qGS.8* | RM256-RM230 | F_2_ |  | |  |  |
| 126 | GW | 9 | *qGW-9* | G1445-XNpb293 | RILs |  | | Asominori/IR24 | [22] |
| 127 | GW | 10 | *qGW-10* | C16-XNpb127 | RILs |  | |  |  |
| 128 | GL | 11 | *qGL.11* | RM437-RM289 | F_2_ |  | | Domsephid/Gerdeh | [17] |
| 129 | GTN | 12 | *qGTN12* | RM19-RM147 | RILs |  | | TD70/Kasalath | [18] |
| 131 | GW | 12 | *qGW-12* | RM235-RM17 | ILs |  | | Teqing/lemont | [13] |
| 132 | GW | 12 | *qGW-12* | R1869-R367 | RILs |  | | Asominori/IR24 | [22] |
| **C** | **CP-Cooking properties** | | | | | | | | |
| 133 | AC | 1 | *qAC.1* | RM243-RM582 | BC_2_F_2_ |  | | *O. nivara*/Swarna | [6] |
| 134 | GC | 1 | *qGC-1-1* | C904-R2632 | RILs |  | | Zhenshan97/Minghui63 | [25] |
| 135 | GC | 2 | *qGC.2* | RM1075-RM2634 | CSSLs |  | | Koshihikari/Nona Bokra | [4] |
| 136 | AC | 2 | *qac2.1* | RM262-RM3515 | BC_2_F_2_ |  | | Swarna/IRGC81848 | [6] |
| 137 | AC | 2 | *qAC.2* | RM525-RM221 | F_8_, RILs |  | | Chuan7/Nanyangzhan | [8] |
| 138 | AC | 2 | *qAC-2* | RM525-RM221 | RILs |  | |  |  |
| 139 | AC | 3 | *qAC-3-1* | RM36-RM7 | F_2_ |  | | Basmati370/MR84 | [20] |
| 140 | AC | 3 | *qAC.3* | RM22-RM7 | BC_2_F_2_ |  | | *O. nivara*/ Swarna | [6] |
| 141 | GC | 4 | *qgc4.1* | RM185-RM241 | BC_2_F_2_ |  | | Swarna/IRGC81832 |  |
| 142 | AC | 4 | *qAC-4-3* | C56-C820 | RILs |  | | Zhenshan97/Minghui63 | [11] |
| 143 | FC | 5 | *qFC-5* | RG480-RM274 | RILs |  | | Xieqingzao B/Milyang | [26] |
| 144 | AC | 5 | *qac5.1* | RM413-RM13 | BC_2_F_2_ |  | | Swarna/IRGC81832 | [6] |
| 145 | AC | 6 | *qAc6* | C1004-R1962 | DHs |  | | Yuefu/IRAT109 | [27] |
| 146 | GT | 6 | *qGt6b* | RM253-M314 | DHs |  | |  |  |
| 147 | AC | 6 | *qamy_6.1* | RM19226-RM190 | RILs | RM19226-RM190 | | L-202/Lemont | [7] |
| 148 | ASV | 6 | *qASV-6-1* | Waxy-C1496 | RILs |  | | Zhenshan97/Minghui63 | [25] |
| 149 | AC | 6 | *qamy6-1* | RM3-RM217 | RILs |  | | Pusa1121/ Pusa1342 | [16] |
| 150 | ASV | 6 | *qasv6-1* | RM3-RM217 | RILs |  | |  |  |
| 151 | AC | 6 | *qamy6-1* | RM3-RM217 | RILs |  | |  |  |
| 152 | AC | 6 | *qAC-6b* | RM588-RM540 | RILs | RM588-RM540 | | Chuan7/Nanyanghan | [8] |
| 153 | AC | 6 | *ac6.1* | RM190 | RILs |  | | Cypress/ Panda | [2] |
| 154 | AC | 6 | *qAC.6* | RM190-RM587 | DHs |  | | H94/ Zhenshan 97 | [28] |
| 155 | AC | 6 | *qAC.6* | RM454-RM3567 | ILs |  | | *O. rufipogon*/*O. sativa* | [29] |
| 156 | GC | 7 | *qGc7* | RM47-RM172 | DHs |  | | Yuefu/IRAT109 | [27] |
| 157 | AC | 8 | *qAC.8* | RM5911-SA1656 | CSSLs |  | | Koshihikari/Nona Bokra | [4] |
| 158 | AC | 9 | *qAml-9* | RM219-RM23914 | DHs |  | | Cheongcheong/ Nagdong | [30] |
| 159 | AC | 11 | *qAc11b* | G181-G320 | DHs |  | | Yuefu/IRAT109 | [27] |
| 160 | GT | 11 | *qgt.11* | RM5599-RM5961 | CSSLs |  | | Koshihikari/Nona Bokra | [4] |
| 161 | AC | 11 | *qAC.11* | RM209-RM229 | DHs |  | | H94/Zhenshan97 | [28] |
| 162 | AC | 12 | *qAC.12* | RM270-RM235 | DHs |  | |  |  |
| **D** | **FRG-Fragrance of rice grain** | | | | | | | | |
| 163 | Aroma | 3 | *qaro3-1* | RM5474-RM282 | RILs |  | | Pusa 1121/Pusa 1342 | [16] |
| 164 | Aroma | 8 | *qAR-8-1* | RM42-RM223 | F_2_ | RM223 | | Basmati 370/MR 84 | [20] |
| 165 | Aroma | 8 | *aro8-1* | RM223-RM80 | RILs | RM223-RM80 | | Pusa 1121/Pusa 1342 | [16] |
| 166 | Aroma | 8 | *qAR-8-4* | RM515-RM210 | F_2_ | RM515-RM210 | |  |  |
| **E** | **NF-Nutrition factors** | | | | | | | | |
| 167 | PC | 1 | *qPr1* | RM493-RM562 | RILs | RM493-RM562 | Zhenshan97B/Delong 208 | | [31] |
| 168 | PC | 1 | *qPC1.1* | 1008-RM575 | DHs |  | Samgang/Nagdong | | [32] |
| 169 | MAC-P | 1 | *qP.1* | RM3411 | LT/TL-RILs |  | TeQing/Lemont | | [15] |
| 170 | MAC-K | 1 | *qK.1* | RM5501 | LT/TL-RILs |  | Lemont/TeQing | |  |
| 171 | PC | 1 | *qPC1* | RM472-RM104 | RILs |  | Zhenshan97/Nanyangzhan | | [11] |
| 172 | AAC | 1 | *qAa1* | RM493-RM562 | RILs |  | Zhenshan97B/Delong 208 | | [31] |
| 173 | MAC-P | 1 | *qP.1* | RM495 | LT/TL-RILs |  | Lemont/TeQing | | [15] |
| 174 | MAC-Cd | 1 | *qCd.1* | RM6840 | LT-RILs |  |  |  |  |
| 175 | Zn | 1 | *qZn.1* | RM34-RM237 | DHs |  | IR64/Azucena | | [33] |
| 176 | Mn | 1 | *qMn.1* | RM243-RM312 | DHs |  |  |  |  |
| 177 | MAC-Co | 1 | *qCo.1* | RM490 | LT/TL-RILs |  | Lemont/Teqing | | [15] |
| 178 | MAC-Ca | 1 | *qCa1-1* | RM6480 | ILs |  | *O. rufipogon*/Teqing | | [34] |
| 179 | MAC-P | 1 | *qP1-1* | RM212 | ILs |  |  |  |  |
| 180 | Fe | 1 | *qFe1.1* | RM243-RM488 | RILs |  | Madhukar/Swarna | | [35] |
| 181 | Fe | 1 | *qFe1.2* | RM488-RM490 | RILs |  |  |  |  |
| 182 | AAC-Asp/ Thr/ Glu/ Gly/ Ala/ Cys/ Tyr/ Pro/ Eaa/ Total | 1 | *qAA.1* | RM472-RM104 | RILs | RM472 | Zhenshan97/Nanyangzhan | | [36] |
| 183 | Fe | 1 | *qFe.1* | RM259-RM243 | RILs | RM259-RM243 | Zhenshan 97/Minghui 63 | | [37] |
| 184 | MIC-Fe | 2 | *qFe2-1* | RM6641 | ILs |  | *O. rufipogon*/Teqing | | [34] |
| 185 | PC | 2 | *qPC-2* | RM5897-RM6247 | RILs |  | Chuan7/Nanyanghan | | [38] |
| 186 | MIC-Cu | 2 | *qCu.2* | RM6378 | LT/TL-RILs |  | Lemont/TeQing | | [15] |
| 187 | MAC-Sr | 2 | *qSr.2* | RM3688 | LT-RILs |  |  |  |  |
| 188 | Fe | 2 | *qFe.2* | RM53-RM300 | DHs | RM53-RM300 | IR64/Azucena | | [33] |
| 189 | AAC-His | 2 | *qAA.2* | RM324-RM301 | RILs | RM301 | Zhenshan97/Nanyangzhan | | [36] |
| 190 | AAC- Val/ Ile/ Leu/ His/ Phe | 2 | *qAA.2* | RM322-RM521 | RILs | RM521 |  |  |  |
| 191 | PC | 2 | *qLip-2* | RM5619-RM1211 | DHs |  | Cheongcheong/Nagdong | | [30] |
| 192 | MIC-Fe | 2 | *qFe.2* | RM452 | LT/TL-RILs |  | Lemont/TeQing | | [15] |
| 193 | MIC-Mn | 2 | *qMn2-1* | RM6367 | ILs |  | *O. rufipogon*/Teqing | | [34] |
| 194 | MAC-S | 2 | *qS.2* | RM266 | LT-RILs |  | Lemont/TeQing | | [15] |
| 195 | MAC-Ca | 3 | *qCa.3* | RM5626-RM16 | LT/TL-RILs |  | Lemont/TeQing  Zhenshan97/Nanyangzhan | | [15, 36] |
| 196 | MAC-Rb | 3 | *qRb.3* | RM489 | LT-RILs |  |  |  |  |
| 197 | AAC-Tyr | 3 | *qAA.3* | RM520-RM468 | RILs | RM520 |  |  |  |
| 198 | MAC-Mg | 3 | *qMg3-1* | RM5488 | ILs |  | O*. rufipogon*/Teqing | | [34] |
| 199 | Ca | 3 | *qCa.3.* | RM200-RM227 | RILs |  | Zhenshan 97/Minghui 63 | | [37] |
| 200 | PC | 3 | *qPC-3* | RM251-RM282 | RILs |  | Xieqingzao B/Milyang | | [26] |
| 201 | Zn | 3 | *qZn3.1* | RM7-RM517 | RILs |  | Madhukar×Swarna | | [35] |
| 202 | PC | 3 | *qPC-3* | RM251-RM282 | RILs |  | Xieqingzao B/Milyang | | [26] |
| 203 | Mn | 3 | *qMn.3* | RM227-R1925 | RILs |  | Zhenshan 97/Minghui 63 | | [37] |
| 204 | Cu | 3 | *qCu.1* | R1925-RM148 | RILs | R1925-RM148 | Zhenshan 97/Minghui 63  Zhenshan97/Nanyangzhan | | [36, 37] |
| 205 | AAC-Thr/ Gly /His/ Arg | 4 | *qAA.4* | RM348-RM131 | RILs | RM131 |  |  |  |
| 206 | CPB | 4 | *qcpb4* | E12M61.256 | RILs |  | Cypress/Panda | | [2] |
| 207 | CPH | 4 | *qcph4* | E12M61.256 | RILs |  | Cypress/Panda  Zhenshan 97/Minghui 63 | | [2, 37] |
| 208 | Cu | 5 | *qCu.5* | C1447-RM31 | RILs |  |  |  |  |
| 209 | PA | 5 | *qPA.5* | RM305-RM178 | DHs |  | IR64/Azucena | | [33] |
| 210 | FC | 5 | *qFC-5* | RG480-RM274 | RILs |  | Xieqingzao B/Milyang | | [26] |
| 211 | Fe | 5 | *qFe5.1* | RM574-RM122 | RILs |  | Madhukar/Swarna | | [35] |
| 212 | MAC-Ca | 5 | *qCa5-1* | RM598 | ILs |  | *O. rufipogon*/Teqing | | [34] |
| 213 | MIC-Zn | 5 |  | RM421 | LT/TL-RILs |  | Lemont/TeQing | | [15] |
| 214 | LC | 6 | *qLIp-6* | RM586-RM1163 | DHs |  | Cheongcheong/Nagdong | | [30] |
| 215 | PC | 6 | *qPC-6* | RM190-RZ516 | RILs | RM190-RZ516 | Xieqingzao B/Milyang | | [26] |
| 216 | FC | 6 | *qFC-6* | RM190-RZ516 | RILs | RM190-RZ516 | Xieqingzao B/Milyang | | [26] |
| 217 | MIC-Cu | 6 | *qCu6-1* | RM204 | ILs |  | O. rufipogon/Teqing | | [34] |
| 218 | Zn | 6 | *qZn.6* | RZ398-RM204 | RILs |  | Zhenshan 97/Minghui 63 | | [37] |
| 219 | PC | 6 | *qPC-6* | RM190-RZ516 | RILs |  | Xieqingzao B/Milyang | | [26] |
| 220 | MAC-Mg | 6 | *qMg.6* | OSR 21 | LT/TL-RILs |  | Lemont/TeQing | | [15] |
| 221 | PC | 7 | *qPc7* | RM270-C751 | DHs |  | Yuefu/IRAT109 | | [27] |
| 222 | MIC-Mn | 7 | *qMn.7* | RM214 | LT/TL-RILs |  | Lemont/TeQing | | [15] |
| 223 | AAC-Pro/ Gly/ Met/ Arg | 7 | *qAA.7* | RM125-RM214 | RILs | RM214 | Zhenshan97/Nanyangzhan | | [36] |
| 224 | Zn | 7 | *qZn7.3* | RM501-OsZip2 | RILs |  | Madhukar/Swarna | | [35] |
| 225 | Fe | 7 | *qFe7.1* | RM234-RM248 | RILs |  | Madhukar/Swarna  IR64/Azucena | | [33, 35] |
| 226 | MAC-P | 7 | *qP.7* | RM70-RM172 | DHs |  |  |  |  |
| 227 | PC | 7 | *qPC.1* | R1245-RM234 | RILs |  | Zhenshan97/Minghui 63 | | [10] |
| 228 | PC | 7 | *qPr7* | RM445-RM418 | RILs |  | Zhenshan97B/Delong 208 | | [31] |
| 229 | MIC-Zn | 8 | *qZn8-1* | RM152 | ILs |  | *O. rufipogon*/Teqing | | [34] |
| 230 | AAC-Tyr | 8 | *qAA.8* | RM137-RM556 | RILs | RM556 | Zhenshan97/Nanyangzhan | | [36] |
| 231 | AAC-Cys | 8 | *qAA.8* | RM447-RM458 | RILs | RM447 | Zhenshan97/Nanyangzhan  *O. rufipogon*/Teqing | | [34, 36] |
| 232 | MAC-K | 8 | *qK8-1* | RM3572 | ILs |  |  |  |  |
| 233 | Zn | 8 | *qZn.8* | RM25-R1629 | RILs |  | Zhenshan 97/Minghui 63 | | [37] |
| 234 | Cu | 8 | *qCu.8* | RM201-C472 | RILs |  | Zhenshan 97/Minghui 63  IR64/Azucena | | [33, 37] |
| 235 | Fe | 8 | *qFe.8* | RM137-RM325A | DHs |  |  |  |  |
| 236 | AAC | 9 | *qAa9* | RM328-RM107 | RILs |  | Zhenshan97B/Delong 208 | | [31] |
| 237 | MAC-P | 9 | *qP9-1* | RM201 | ILs |  | *O. rufipogon*/Teqing | | [34] |
| 238 | MAC-Mg | 10 | *qMg.10* | RM467 | LT-RILs |  | Lemont/TeQing | | [15] |
| 239 | AAC-Cys/ Leu/ Ile/ Phe | 10 | *qAA.10* | RM467-RM271 | RILs | RM271 | Zhenshan97/Nanyangzhan | | [36] |
| 240 | PC | 10 | *qPC-10* | RM184-RM3229B | RILs |  | Xieqingzao B/Milyang | | [26] |
| 241 | PC | 10 | *qPro-10* | RM24934-RM25128 | DHs | RM24934 | Cheongcheong/ Nagdong | | [30] |
| 242 | MAC-Mg | 11 | *qMg.11* | RM332 | LT/TL-RILs |  | Lemont/TeQing | | [15] |
| 243 | MIC-Cu | 11 | *qCu.11* | RM167 | LT-RILs |  | Lemont/TeQing  Samgang and Nagdong | | [15, 32] |
| 244 | PC | 11 | *qPC1.11* | 1027-RM287 | DHs | RM287 |  |  |  |
| 245 | Fe | 11 | *qFe.11* | RZ536-TEL3 | RILs |  | Zhenshan 97/Minghui 63 | | [37] |
| 246 | PC | 11 | *qPC1.11* | RM287-RM26755 | DHs | RM287 | Samgang and Nagdong | | [32] |
| 247 | PA | 12 | *qPA.12* | RM247-RM179 | DHs |  | IR64/Azucena | | [33] |
| 248 | Fe | 12 | *qFe.12* | RM270-RM17 | DHs |  | IR64/Azucena  Madhukar/Swarna | | [11, 33] |
| 249 | Zn | 12 | *qZn.12* | RM235-RM17 | DHs |  |  |  |  |
| 250 | Fe | 12 | *qFe12.2* | RM260-RM7102 | RILs |  |  |  |  |
| 251 | Fe | 12 | *qFe12.1* | RM17-RM260 | RILs |  | Madhukar/Swarna | | [35] |
| 252 | Zn | 12 | *qZn12.2* | RM260-RM7102 | RILs |  |  |  |  |

**[(A) MPGQ = Milling properties of grain quality;** **MP**- Milling percentage; **HR**-Head rice (%);**BR-** Brown rice (%); **PGWC**- percentage of grain with white core; **RB**-Rice bran (%); **MRP**-milled rice percentage; **MHP**-milled head rice percentage; **BRP**-Brown rice percentage; **BRR**- Brown rice recovery; **MRR**-Milled rice recovery; **HRR**-head rice recovery; **VER**- Volume expansion ratio; **KL**- Kernel length (mm); **KLC**- Kernel length after cooking (mm); **CR**- chalkiness rate; **CA**- chalkiness area; **CS**- chalkiness score; **DC**-Degree of chalkiness; **KLB**- Kernel length brown rice (mm); **KW**- Kernel weight; **KLH**- Kernel length head rice (mm); **KWB**- Kernel width brown rice (mm); **KTN**-Kernal thickness; **(B)** **GA = Grain appearance; GL**- Grain length; **GW**- Grain width; **GB**-Grain breadth; **GS**-Grain size; **GTN**-Grain thickness; **GV**-Grain volume; **GC**- Grain chalkiness; **PGC**-percentage of grains with chalkiness; **GE**- Grain elongation; **LBR**- Length/ breadth ratio; **GS**- Grain size; **LW**- Grain length–width ratio; **GCF**-Grain circumference; **GSW**- Grain size and weight; **(C)** **CP = Cooking properties; AC**-Amylose content; **GC**-Gel consistency; **GT**-Gelatinization temperature; **ASV**-Alkaline spreading value; **FRG = Fragrance of rice grain; AR-**Aroma; **(D) NF = Nutrition factors; PC**- Protein content; **PA**-Phytic acid; **AAC**-Amino acid content; **CPB**- Crude protein brown rice; **CPH**- Crude protein head rice **MIC**- Micro-element; **MAC**- Macro-element; **LC**-Lipid content; **FC**- Fat content]

**References**

1. G. Aluko, C. Martinez, J. Tohme, C. Castano, C. Bergman, and J.H.H. Oard: “QTL mapping of grain quality traits from the interspecific cross Oryza sativa× O. glaberrima.” *Theor. Appl. Genet.* vol. 109, no. 3, pp. 630–639, 2004.

2. J.L. Kepiro, A.M. McClung, M.H. Chen, K.M. Yeater, and R.G. Fjellstrom: “Mapping QTLs for milling yield and grain characteristics in a tropical japonica long grain cross.” *J. Cereal Sci.* vol. 48, no. 2, pp. 477–485, 2008.

3. E.M. Septiningsih, K.R. Trijatmiko, S. Moeljopawiro, S.R. McCouch, E.M. Eptiningsih, and K.R. Trijatmiko: “Identification of quantitative trait loci for grain quality in an advanced backcross population derived from the Oryza sativa variety IR64 and the wild relative O. rufipogon.” *Theor Appl Genet*. vol. 107, no. 8, pp. 1433–1441, 2003.

4. W. Hao, M. Zhu, J. Gao, S. Sun, and H. Lin: “Identification of quantitative trait loci for rice quality in a population of chromosome segment substitution lines.” *J. Integr. Plant Biol.* vol. 51, no. 5, pp. 500–512, 2009.

5. J.C. Nelson, F. Jodari, A.I. Roughton, K.M. Mckenzie, A.M. Mcclung, R.G. Fjellstrom, and B.E. Scheffler: “QTL mapping for milling quality in elite western US rice germplasm.” *Crop Sci.* vol. 52, no. 1, pp. 242–252, 2012.

6. B.P.M.M. Swamy, K. Kaladhar, N.S. Rani, G.S.V. V Prasad, B.C. Viraktamath, G.A. Reddy, N. Sarla, N. Shobha Rani, G.S.V. V Prasad, B.C. Viraktamath, G.A. Reddy, and N. Sarla: “QTL analysis for grain quality traits in 2 BC2F2 populations derived from crosses between Oryza sativa cv Swarna and 2 accessions of O. nivara.” *J. Hered.* vol. 103, no. 3, pp. 442–452, 2012.

7. J.C. Nelson, A.M. Mcclung, R.G. Fjellstrom, K.A.K.K. Moldenhauer, E. Boza, F. Jodari, J.H. Oard, S. Linscombe, B.E. Scheffler, and K.M. Yeater: “Mapping QTL main and interaction influences on milling quality in elite US rice germplasm.” *Theor. Appl. Genet.* vol. 122, no. 2, pp. 291–309, 2011.

8. A. Anandan, T. Sabesan, R. Eswaran, G. Rajiv, N. Muthalagan, and R. Suresh: “Appraisal of environmental interaction on quality traits of rice by additive main effects and multiplicative interaction analysis.” *Cereal Res. Commun.* vol. 37, no. 1, pp. 131–140, 2009.

9. Y. Dong, E. Tsuzuki, D. Lin, H. Kamiunten, H. Terao, M. Matsuo, and S. Cheng: “Molecular genetic mapping of quantitative trait loci for milling quality in rice (Oryza sativa L.).” *J. Cereal Sci.* vol. 40, no. 2, pp. 109–114, 2004.

10. Y.F. Tan, M. Sun, Y.Z. Xing, J.P. Hua, X.L. Sun, Q.F. Zhang, and H. Corke: “Mapping quantitative trait loci for milling quality, protein content and color characteristics of rice using a recombinant inbred line population derived from an elite rice hybrid.” *Theor. Appl. Genet.* vol. 103, no. 6–7, pp. 1037–1045, 2001.

11. B. Peng, L. Wang, C. Fan, G. Jiang, L. Luo, Y. Li, and Y. He: “Comparative mapping of chalkiness components in rice using five populations across two environments.” *BMC Genet.* vol. 15, no. 1, pp. 49, 2014.

12. P.R. Chandusingh, N.K. Singh, K. V Prabhu, K.K. Vinod, and A.K. Singh: “Molecular mapping of quantitative trait loci for grain chalkiness in rice (Oryza sativa L.).” *Indian J. Genet. Plant Breed*. vol. 73, no. 3, pp. 244–251, 2013.

13. T.Q. Zheng, J.L. Xu, Z.K. Li, H.Q. Zhai, and J.M. Wan: “Genomic regions associated with milling quality and grain shape identified in a set of random introgression lines of rice (Oryza sativa L.).” *Plant Breed.* vol. 126, no. 2, pp. 158–163, 2007.

14. C. Yin, H. Li, S. Li, L. Xu, Z. Zhao, and J. Wang: “Genetic dissection on rice grain shape by the two-dimensional image analysis in one japonica × indica population consisting of recombinant inbred lines.” *Theor. Appl. Genet.* vol. 128, no. 10, pp. 1969–1986, 2015.

15. M. Zhang, S.R.M. Pinson, L. Tarpley, X.-Y. Huang, B. Lahner, E. Yakubova, I. Baxter, M. Lou Guerinot, and D.E. Salt: “Mapping and validation of quantitative trait loci associated with concentrations of 16 elements in unmilled rice grain.” *Theor. Appl. Genet.* vol. 127, no. 1, pp. 137–165, 2014.

16. Y. Amarawathi, R. Singh, A.K. Singh, V.P. Singh, T. Mohapatra, T.R. Sharma, and N.K. Singh: “Mapping of quantitative trait loci for basmati quality traits in rice (Oryza sativa L.).” *Mol. Breed.* vol. 21, no. 1, pp. 49–65, 2008.

17. B. Rabiei, M. Valizadeh, B. Ghareyazie, M. Moghaddam, and A.J. Ali: “Identification of QTLs for rice grain size and shape of Iranian cultivars using SSR markers.” *Euphytica*. vol. 137, no. 3, pp. 325–332, 2004.

18. Y. ZHANG, Y. ZHANG, S. DONG, C. Tao, Q. ZHAO, Z.H.U. Zhen, L. ZHOU, Y.A.O. Shu, Z. Ling, and Y.U. Xing: “QTL mapping for grain size traits based on extra-large grain rice line TD70.” *Rice Sci.* vol. 20, no. 6, pp. 400–406, 2013.

19. X.-J. Song, W. Huang, M. Shi, M.-Z. Zhu, and H.-X. Lin: “A QTL for rice grain width and weight encodes a previously unknown RING-type E3 ubiquitin ligase.” *Nat. Genet.* vol. 39, no. 5, pp. 623, 2007.

20. A. Cheng, I. Ismail, M. Osman, and H. Hashim: “Mapping of quantitative trait loci for aroma, amylose content and cooked grain elongation traits in rice.” *Plant Omics*. vol. 7, no. 3, pp. 152, 2014.

21. Y.F. Tan, Y.Z. Xing, J.X. Li, S.B. Yu, C.G. Xu, and Q. Zhang: “Genetic bases of appearance quality of rice grains in Shanyou 63, an elite rice hybrid.” *Theor. Appl. Genet.* vol. 101, no. 5–6, pp. 823–829, 2000.

22. X. Wan, J. Weng, H. Zhai, J. Wang, C. Lei, X. Liu, T. Guo, L. Jiang, N. Su, and J. Wan: “QTL analysis for rice grain width and fine mapping of an identified QTL allele gw-5 in a recombination hotspot region on chromosome 5.” *Genetics*. vol. 179, no. 4, pp. 2239–2252, 2008.

23. A. Shomura, T. Izawa, K. Ebana, T. Ebitani, H. Kanegae, S. Konishi, and M. Yano: “Deletion in a gene associated with grain size increased yields during rice domestication.” *Nat. Genet.* vol. 40, no. 8, pp. 1023, 2008.

24. R. Singh, A.A.K. Singh, T.R. Sharma, A.A.K. Singh, and N.K. Singh: “Fine mapping of grain length QTLs on chromosomes 1 and 7 in Basmati rice (Oryza sativa L.).” *J. plant Biochem. Biotechnol.* vol. 21, no. 2, pp. 157–166, 2012.

25. X. Zheng, J.G. Wu, X.Y. Lou, H.M. Xu, and C.H. Shi: “The QTL analysis on maternal and endosperm genome and their environmental interactions for characters of cooking quality in rice (Oryza sativa L.).” *Theor. Appl. Genet.* vol. 116, no. 3, pp. 335–342, 2008.

26. Y.-H.H. Yu, G. Li, Y.-Y.Y. Fan, K.-Q.Q. Zhang, J. Min, Z.-W.W. Zhu, and J.-Y.Y. Zhuang: “Genetic relationship between grain yield and the contents of protein and fat in a recombinant inbred population of rice.” *J. Cereal Sci.* vol. 50, no. 1, pp. 121–125, 2009.

27. Y. Guo, P. Mu, J. Liu, Y. Lu, and Z. Li: “QTL mapping and Q× E interactions of grain cooking and nutrient qualities in rice under upland and lowland environments.” *J. Genet. Genomics*. vol. 34, no. 5, pp. 420–428, 2007.

28. C. Fan, Y. Xing, H. Mao, T. Lu, B. Han, C. Xu, X. Li, and Q. Zhang: “GS3, a major QTL for grain length and weight and minor QTL for grain width and thickness in rice, encodes a putative transmembrane protein.” *Theor. Appl. Genet.* vol. 112, no. 6, pp. 1164–1171, 2006.

29. P.-R. Yuan, H.-J. Kim, Q.-H. Chen, H.-G. Ju, S.-D. Ji, and S.-N. Ahn: “Mapping QTLs for grain quality using an introgression line population from a cross between Oryza sativa and O. rufipogon.” *J. Crop Sci. Biotechnol.* vol. 13, no. 4, pp. 205–212, 2010.

30. B.B.-W. Yun, M.-G.M. Kim, T. Handoyo, and K.K.-M. Kim: “Analysis of rice grain quality-associated quantitative trait loci by using genetic mapping.” *Am. J. Plant Sci.* vol. 5, no. 09, pp. 1125, 2014.

31. M. ZHONG, L. qiang Wang, D. jun YUAN, L. jun Luo, C. guo Xu, and Y. qing HE: “Identification of QTL affecting protein and amino acid contents in rice.” *Rice Sci.* vol. 18, no. 3, pp. 187–195, 2011.

32. Y. Qin, S.-M. Kim, and J.-K. Sohn: “QTL analysis of protein content in double-haploid lines of rice.” *KOREAN J. Crop Sci.* vol. 54, no. 2, pp. 165–171, 2009.

33. J.C.R.R. Stangoulis, B.-L.L. Huynh, R.M. Welch, E.-Y.Y. Choi, and R.D. Graham: “Quantitative trait loci for phytate in rice grain and their relationship with grain micronutrient content.” *Euphytica*. vol. 154, no. 3, pp. 289–294, 2007.

34. A.L. Garcia-Oliveira, L. Tan, Y. Fu, C. Sun, A.L. Garcia‐Oliveira, L. Tan, Y. Fu, and C. Sun: “Genetic identification of quantitative trait loci for contents of mineral nutrients in rice grain.” *J. Integr. Plant Biol.* vol. 51, no. 1, pp. 84–92, 2009.

35. K. Anuradha, S. Agarwal, Y.V. Rao, K. V Rao, B.C. Viraktamath, and N. Sarla: “Mapping QTLs and candidate genes for iron and zinc concentrations in unpolished rice of Madhukar× Swarna RILs.” *Gene*. vol. 508, no. 2, pp. 233–240, 2012.

36. L. Wang, M. Zhong, X. Li, D. Yuan, Y. Xu, H. Liu, Y. He, L. Luo, and Q. Zhang: “The QTL controlling amino acid content in grains of rice (Oryza sativa) are co-localized with the regions involved in the amino acid metabolism pathway.” *Mol. Breed.* vol. 21, no. 1, pp. 127–137, 2008.

37. K. Lu, L. Li, X. Zheng, Z. Zhang, T. Mou, and Z. Hu: “Quantitative trait loci controlling Cu, Ca, Zn, Mn and Fe content in rice grains.” *J. Genet.* vol. 87, no. 3, pp. 305–310, 2008.

38. J. Lou, L. Chen, G. Yue, Q. Lou, H. Mei, L. Xiong, and L. Luo: “QTL mapping of grain quality traits in rice.” *J. Cereal Sci.* vol. 50, no. 2, pp. 145–151, 2009.
